# Is there an optimal minimally invasive technique for left anterior descending coronary artery bypass?

**DOI:** 10.1186/1749-8090-6-37

**Published:** 2011-03-25

**Authors:** Olivier Jegaden, Fabrice Wautot, Thomas Sassard, Isabella Szymanik, Abdel Shafy, Joel Lapeze, Fadi Farhat

**Affiliations:** 1 Department of cardiac surgery and transplantation, Hospital Louis Pradel, University Claude Bernard Lyon1, INSERM 886, 59 Boulevard Pinel, 69677 Bron France

## Abstract

**Background:**

The aim of this retrospective study was to evaluate the clinical outcome of three different minimally invasive surgical techniques for left anterior descending (LAD) coronary artery bypass grafting (CABG): Port-Access surgery (PA-CABG), minimally invasive direct CABG (MIDCAB) and off-pump totally endoscopic CABG (TECAB).

**Methods:**

Over a decade, 160 eligible patients for elective LAD bypass were referred to one of the three techniques: 48 PA-CABG, 53 MIDCAB and 59 TECAB. In MIDCAB group, Euroscore was higher and target vessel quality was worse. In TECAB group, early patency was systematically evaluated using coronary CT scan. During follow-up (mean 2.7 ± 0.1 years, cumulated 438 years) symptom-based angiography was performed.

**Results:**

There was no conversion from off-pump to on-pump procedure or to sternotomy approach. In TECAB group, there was one hospital cardiac death (1.7%), reoperation for bleeding was higher (8.5% vs 3.7% in MIDCAB and 2% in PA-CABG) and 3-month LAD reintervention was significantly higher (10% vs 1.8% in MIDCAB and 0% in PA-CABG). There was no difference between MIDCAB and PA-CABG groups. During follow-up, symptom-based angiography (n = 12) demonstrated a good patency of LAD bypass in all groups and 4 patients underwent a no LAD reintervention. At 3 years, there was no difference in survival; 3-year angina-free survival and reintervention-free survival were significantly lower in TECAB group (TECAB, 85 ± 12%, 88 ± 8%; MIDCAB, 100%, 98 ± 5%; PA-CABG, 94 ± 8%, 100%; respectively).

**Conclusions:**

Our study confirmed that minimally invasive LAD grafting was safe and effective. TECAB is associated with a higher rate of early bypass failure and reintervention. MIDCAB is still the most reliable surgical technique for isolated LAD grafting and the least cost effective.

## Background

For several decades, left internal thoracic artery (LITA) bypass grafting has been recognised as the gold standard for left anterior descending coronary artery (LAD) revascularization and its beneficial impact was demonstrated in conventional coronary artery bypass grafting (CABG). During the past decade, minimally invasive (MI) CABG, based on the lack of sternotomy approach, has been developed according to the evolution of technology and dedicated surgical tools, and it has been mainly performed in isolated LAD bypass grafting. Nowadays, MI-CABG brings together different surgical concepts: Port-Access surgery (PA-CABG) based on on-pump CABG with mini-thoracotomy and hand-sewn anastomosis, minimally invasive direct coronary artery bypass (MIDCAB) based on off-pump CABG with mini-thoracotomy and hand-sewn anastomosis, and totally endoscopic coronary artery bypass grafting (TECAB) based on on-pump or off-pump surgery with robotic assisted anastomosis. These MI-CABG techniques have been compared to conventional CABG according to observational research because of the lack of randomized trial: the expected clinical results must be at worst as good as conventional CABG; the observational results are at best the same [1-3]. In the same way, these different MI-CABG techniques have never been compared together. In this series, we report our experience in minimally invasive LAD grafting with a comparative analysis between PA-CABG, MIDCAB and off-pump TECAB, in order to answer the question: is there an optimal minimally invasive technique for isolated LAD grafting?

## Methods

From January 1998 to December 2008, 160 eligible patients for elective LAD revascularization were referred to minimally invasive CABG surgery. There were 135 males and 25 females, mean age was 58 ± 11 years and Euroscore 1.7 ± 1.7. There were two surgical periods: from January1998 to September 2003, the intend-to-treat surgical procedure was either PA-CABG or MIDCAB depending on the patient's condition, and after the purchase of standard Da Vinci robotic system in September 2003, the intend-to-treat surgical procedure was either off-pump TECAB or robotic enhanced MIDCAB depending on the patient's condition. In this series, the patients were categorized into three groups according to the surgical procedure performed: PA-CABG (n = 48), MIDCAB (n = 53), TECAB (n = 59).

### Port-Access group

The surgical technique was previously reported [3]. The video-assisted LITA harvesting was done through the anterior left mini-thoracotomy. The femoral approach for cardiopulmonary bypass was used and aortic clamping was done with the Endo-Aortic Clamp™ according to the concept of Port Access™ EndoCPB system. The coronary anastomosis was performed under direct vision with 8/0 Prolene running suture. PA-CABG was the intend-to-treat procedure in 48 patients free of peripherial atherosclerotic disease and it was done in all cases.

### TECAB group

Off-pump TECAB was the intend-to-treat procedure in 78 cases with an auspicious anatomic condition based on preoperative angiography; however, 19 patients had a conversion to MIDCAB during the procedure and a complete TECAB surgery was performed in 59 patients. The TECAB surgical technique was previously reported [4] and intra-coronary shunt was used in all cases. Before January 2005, proximal and distal LAD occlusions were performed using 4-0 ePTFE sutures (n = 11), after only proximal LAD occlusion was done (n = 48). Before July 2006, the anastomosis was done with 8-0 ePTFE running suture (n = 22), after it was done with nitinol Uclips™ as interrupted sutures (n = 37).

### MIDCAB group

MIDCAB was the intend-to-treat procedure as an alternative to PA-CAB in 17 patients with peripherial atherosclerotic disease. Robotic enhanced MIDCAB was the intend-to-treat procedure as an alternative to TECAB in 17 patients with an unfavorable anatomic condition and in 19 patients it was performed as a conversion of an intend-to-treat TECAB procedure (Table 1). In the first sub-group of 17 patients, the video-assisted LITA harvesting was done through the anterior left mini-thoracotomy; myocardial stabilization was obtained with an Estech stabilizer; a proximal LAD occlusion was performed using 4-0 ePTFE sutures; the anastomosis was done under direct vision with intra-coronary shunt and 8-0 Prolene running suture. In patient with robotic enhanced MIDCAB (n = 36), after endoscopic LIMA harvesting, the pericardium was opened, the target vessel was identified, and the left anterior mini-thoracotomy was performed in the ideal position in front of the target anastomotic site. Myocardial stabilization was done using an Octopus TE endoscopic stabilizer (Medtronic Inc.), placed either before or after the mini-thoracotomy, and the anastomosis was performed according to the same rules with proximal LAD occlusion, intra-coronary shunt and running suture.

**Table 1:** Indications for robotic enhanced MIDCAB (n = 36)

	Intent-to-treat MIDCAB N = 17	Conversion from TECAB N = 19
Quality of LAD	9	8

Sequential graft indication	6	-

Intra-myocardial LAD	1	3

Pleural adhesions	-	3

Stabilizer failure	-	2

Limited anterior space	-	2

Septal back flow	-	1

Unstable angina	1	-

LAD, Left anterior descending coronary artery; MIDCAB, minimally invasive direct coronary artery bypass grafting; TECAB, totally endoscopic coronary artery bypass grafting. Note that all conversions except one (septal back flow) were decided before the anastomosis stage.

All the operations were performed with the patient's informed consent. Data was prospectively collected. Early patency was evaluated by an angiography or CT scan, systematically in the TECAB group, and only in cases of sequential LIMA graft or post-operative troponin level rise in other groups. All the patients had a stress ECG test during the 2-month post-operative period according to the rehabilitation protocol. Follow-up was made by mail enquiries and completed for all patients. During follow-up, only symptom-based coronary angiography was performed. Mean follow-up was 2.7 ± 0.1 years and cumulated follow-up was 438 years.

Data was accessed and analysed with statistical software. Categorial variables are expressed as number and percentage of patients and were analysed with the Fischer exact test or χ² test. Continuous variables were compared with a two-samples t-test. A log-rank test was used to compare Kaplan-Meier curves of survival and freedom from event.

## Results

Patient populations were almost similar in the three groups (Table 2). However, in the MIDCAB group, the Euroscore was significantly higher, related to the significantly higher amount of females; in the TECAB group, sequential LITA graft to LAD and diagonal artery was significantly lower, related to the selection of the indications in this group (Table 1). In any group, there was no conversion from off-pump to on-pump procedure or to sternotomy approach. There was no difference in intervention time and complete revascularization between groups (Table 2). Intubation time was significantly lower after TECAB without any differences between PA-CABG and MIDCAB (Table 3). There was no difference between groups regarding ICU stay, Troponin level and blood loss.

**Table 2:** Preoperative and intraoperative data

	PA-CABG N = 48	MIDCAB N = 53	TECAB N = 59
Mean age (years)	55 ± 9	61 ± 8	59 ± 12

Gender (M/F)	44/4	38/15 *	53/6

Angina CCSC (mean)	3 ± 0.3	2.9 ± 0.4	3 ± 0.3

LVEF (%)	58 ± 11	59 ± 8	57 ± 8

Euroscore	0.9 ± 1	2.3 ± 1.7 *	1.3 ± 1.6

Previous PCI	10 (21)	10 (19)	11 (18)

CPB time (min)	52 ± 15	-	-

Aortic clamp time (min)	34 ± 15	-	-

Intervention time (hrs)	3.2 ± 0.6	3.1 ± 0.7	3.4 ± 0.7

Sequential LAD+Diag	7 (15)	10 (19)	3 (5) *

Complete revascularization	35 (73)	38 (72)	42 (71)

Number of patients with (%); PA-CABG, Port-Access coronary artery bypass grafting; MIDCAB, minimally invasive direct coronary artery bypass grafting; TECAB, totally endoscopic coronary artery bypass grafting; CCSC, Canadian Cardiovascular Society Classification; LVEF, left ventricular ejection fraction; PCI, percutaneous coronary intervention; CPB, cardio-pulmonary bypass; LAD, left anterior descending artery; Diag, diagonal artery. * p < 0.05.

**Table 3:** Early Postoperative results

	PA-CABG N = 48	MIDCAB N = 53	TECAB N = 59
Intubation time (hrs)	8 ± 4	7.2 ± 5.6	4.6 ± 2.4 *

ICU stay (days)	1.7 ± 2.7	1 ± 1.3	0.96 ± 0.8

Troponin (24 hrs, IU)	1.7 ± 2.4	2.1 ± 5	2.2 ± 10

Drainage (24 hrs, ml)	377 ± 245	408 ± 174	368 ± 159

Reoperation for bleeding	1 (2)	2 (3.7)	5 (8.5)

MI	1 (2)	0	2 (3.4)

Stroke	1 (2)	0	0

Reintervention	0	1 (1.8)	4 (6.8) *

Hospital stay (days)	7 ± 3	6.5 ± 1.5	5.5 ± 1.6 *

30-day mortality	0	0	1 (1.7)

Number of patients with (%); PA-CABG, Port-Access coronary artery bypass grafting; MIDCAB, minimally invasive direct coronary artery bypass grafting; TECAB, totally endoscopic coronary artery bypass grafting; ICU, intensive care unit; MI, myocardial infarction. * p < 0.05.

### Early post-operative outcome

Reoperation for bleeding was needed in eight patients: in one MIDCAB patient it was related to the anastomosis which was performed again as an early reintervention; in all other cases, only thoracic wall hemostasis was done and the bleeding source was not always found. In the TECAB group, there was a higher rate of reoperation for bleeding (8.5%) and a mini-thoracotomy was necessary to remove the blood clots in four patients (Table 3).

Post-operative myocardial infarction occurred in one PA-CABG patient, related to a septal artery occlusion and in two TECAB patients, related to an anastomosis dysfunction as demonstrated by angiography.

In the PA-CABG group, only two patients had a post-operative angiography control; in both cases, graft and anastomosis were patent with an occluded septal artery in 1 case. In the MIDCAB group, 13 patients had a postoperative control using either an angiography or CT scan, showing a 100% patency of grafts and anastomoses. In the TECAB group, two patients with postoperative myocardial infarction had angiography control showing anastomosis or post-anastomosis high-grade stenosis; all other patients had a CT scan control before discharge showing an asymptomatic LITA graft occlusion with patent anastomosis in 2 patients, confirmed by angiography. In these 4 patients a reintervention was successfully performed, using stenting done through the native coronary network or the LITA graft. In the TECAB group the patency rate was 93.2% (55/59) and reintervention (6.8%) was significantly higher (Table 3). One PA-CABG patient had a transient postoperative stroke. One TECAB patient died from arythmia after myocardial infarction despite reintervention. Hospital stay was significantly shorter in the TECAB group (Table 3).

### Late post-operative outcome

There was a significant difference in follow-up between the three groups, related to the two surgical periods (Table 4).

**Table 4:** Late postoperative results

	PA-CABG	MICAB	TECAB
Mean follow-up (years)	3.9 ± 0.3 *	2.5 ± 0.3 *	1.8 ± 0.1 *

3-year survival (%)	100	98 ± 5	96 ± 5

3-year angina-free survival (%)	94 ± 8	100	85 ± 12 *

3-year reintervention-free survival (%)	100	98 ± 5	88 ± 8 *

PA-CABG, Port-Access coronary artery bypass grafting; MIDCAB, minimally invasive direct coronary artery bypass grafting; TECAB, totally endoscopic coronary artery bypass grafting; * p < 0.05

In the PA-CABG group, there was no late death. Inferior myocardial infarction occurred in one patient and four patients had a recurrent angina (mean 4 ± 1.4 years postoperatively). In these 5 patients, coronary angiography demonstrated that the event was not related to the LAD bypass and two of them underwent a reintervention: 1 stenting on the right coronary artery and 1 surgical bypass to marginal and posterior descending coronary arteries. At follow-up, the CCS functional class was 1.1 ± 0.3.

In the MIDCAB group, there were two late deaths from cancer (5 months and 8 years post-operatively). One patient had recurrent angina at 7-year post-operatively; coronary angiography demonstrated that the event was not related to the LAD bypass and the patient underwent a surgical reintervention to the marginal and right coronary arteries. At follow-up, the CCS functional class was 1.2 ± 01.4.

In the TECAB group, one patient committed suicide 6 months after surgery. Two patients had recurrent angina during the rehabilitation period (1 and 2 months post-operatively). Coronary angiography demonstrated that the event was related to the LAD bypass ( 1 occlusion of LITA, 1 post-anastomotic stenosis); both patients underwent a stenting of LAD. Six other patients had late recurrent angina (from 1 to 4 years post-operatively); in all these cases, coronary angiography demonstrated that the event was not related to the LAD bypass and one patient had a stenting of the right coronary artery. At follow-up, the CCS functional class was 1.1 ± 0.3.

At 3-year, there was no difference in survival between the three groups. However, 3-year angina-free survival and reintervention-free survival were significantly lower in the TECAB group (Table 4).

## Discussion

Our study confirms minimally invasive CABG, regardless the technique used, is safe with a 0.6% early mortality, and effective with a 98 ± 2% 5-year survival, a 93 ± 6% 5-year freedom from reintervention and a 85 ± 9% 5-year freedom from angina. The early patency of LITA to LAD (94%, 77/82) is comparable to those of conventional on-pump CABG (91%) according to IMAGE trial [5] or off-pump CABG (92%) according to randomized trial [6]. All procedures were performed without conversion from off-pump to on-pump procedure or to sternotomy approach, and all LAD bypass failures could be treated by stenting.

We have analysed MI-CABG results between three different techniques developed during the past decade. PA-CABG is the most sophisticated procedure involving on-pump surgery and endo-aortic clamping technique. In this group, we have observed very satisfactory early and late results, without any post-operative major event; symptom-based angiography demonstrated good graft and anastomosis patency in all cases. Results are comparable to those previously reported with this technique [7,8].

MIDCAB is the less demanding procedure and has gained widespread acceptance according to excellent results provided [9,10], which our series has confirmed. Only one case of reintervention occurred, related to early anastomosis bleeding. We have observed no differences in results between classical and robotic-enhanced MIDCAB. In this study, there was no difference in operative risks and mid-term results between PA-CABG and MIDCAB.

**Figure 1:**
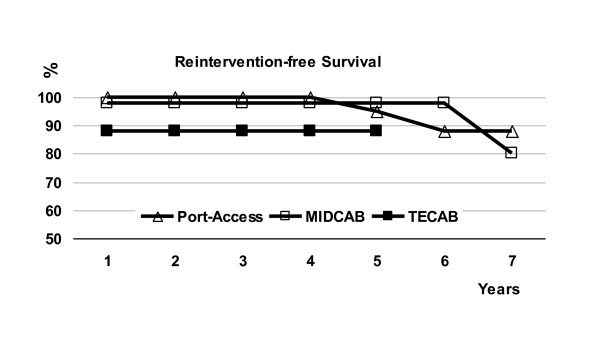
**Actuarial reintervention-free survival according to the surgical technique performed.** PA-CABG, Port-Access coronary artery bypass grafting; MIDCAB, minimally invasive direct coronary artery bypass grafting; TECAB, totally endoscopic coronary artery bypass grafting; p = 0.02 between TECAB and the two other techniques.

TECAB is controversial [2]; off-pump TECAB is the less invasive concept in LAD grafting, nevertheless results are not as good as expected. De Canniere [11] reported a 2.2% early mortality, a 92.1% early patency and a 4.1% reintervention rate at 30 days. In our series, early mortality was 1.7%, early patency was 93.2% and the reintervention rate before discharge was 6.8%. Two more patients underwent reintervention of LAD, 1 and 2 months postoperatively, after symptom-related angiography which showed LAD bypass dysfunction undetected by coronary CT scan before discharge. The actuarial freedom from angina and from reintervention were significantly lower in the TECAB group (Figure 1); it was directly related to a primary bypass failure which remains the main concern in the TECAB procedure. In our experience, modifications of the anastomosis technique allowed to improve the patency: after the occurrence of post-anastomotic dysfunction cases, distal LAD occlusion during anastomosis was abandoned and this type of failure disappeared; anastomotic dysfunction disappeared also when we changed from running suture to uclips suture which provided a 100% patency. Nevertheless, we have observed three cases of LITA occlusion with an opened LAD anastomosis: one seemed to be related to a twist of the graft, the two others remained unexplained. However, the rate of graft failure in the TECAB procedure is acceptable in comparison with classical coronary surgery; routine intraoperative completion angiography in classical CABG demonstrated that 7% of LAD-LITA grafts had a significant defect: 3% in the conduit and 4% at the distal anastomosis [12]. In our study, comparison of the patency between groups was not relevant because systematic assessment was not done in all groups; but there is no question regarding the end-point of LAD reintervention at 3 months (PAC-CAB, 0%; MIDCAB, 1.8%; TECAB, 10%; p = 0.01). Nevertheless, there was no difference in mortality and survival between the three groups.

In all, reoperation for bleeding was high, specially in the TECAB group, demonstrating hemostasis is difficult in a minimally invasive environment and more in a closed chest procedure. In this series, from patients with an intent-to-treat TECAB procedure (n = 78), 24% had a conversion to MIDCAB procedure and from patients who underwent a TECAB procedure (n = 58), 7% had a thoracotomy during reoperation for bleeding. Nevertheless, hospital stay was significantly shorter in TECAB group.

There is no evidence in published data that on-pump TECAB (or Port-Access TECAB) procedure provides better results. In the multicenter European trial [11], there was no difference in 6-month freedom from MACE between on-pump and off-pump TECAB procedures; in the on-pump TECAB multicenter US trial [13], 3-month freedom from reintervention or angiography failure was 91% versus 90% in our series. Better results could be expected with the fourth arm Da Vinci system with the advantage of the robotic endostabilizer.

Our study has its limitations. Patients were not randomized and they were referred to one of the three MI techniques according to the evolution of the MI surgical concept in our team, to their condition and the quality of the target vessel. Inclusion in the MIDCAB group of conversions from an intent-to-treat TECAB procedure is also open to criticism; it was reasonable because all conversions except one were decided before the anastomosis stage and were mainly related to the quality of the LAD. All these bias contributed to include in the MIDCAB group the "worst" cases regarding target vessel quality, which did not have any impact on results, as good as in PA-CABG group and better than in TECAB group. An intention to treat analysis would provide the same results. A systematic post-operative assessment of LAD bypass was performed only after TECAB procedure and patency comparison between groups was not relevant. In any case, the correlation between LAD bypass failure and recurrent angina is well known; in this study, all patients with angina recurrence underwent coronary angiography and comparison between groups was focused on reintervention events.

In conclusion, our study has confirmed minimally invasive CABG is safe and effective. If PA-CABG and MICAB provide results as good as conventional CABG, TECAB procedure is associated with a higher early rate of bypass failure and reintervention. Beyond the post-operative period, results are equivalent and stable regardless the surgical technique performed. According to these results, PA-CABG was abandoned considering its cost effectiveness [8] and patients for LAD grafting are referred either to robotic-enhanced MIDCAB or off-pump TECAB, mainly according to the quality of the target; but in any case of doubt or technical difficulty we don't hesitate to convert before the anastomosis stage, an intent-to-treat TECAB procedure to a MIDCAB procedure which remains the reference procedure for minimally invasive LAD grafting.

## Competing interests

The authors declare that they have no competing interests.

## Authors' contributions

OJ conceived of the study, and drafted the manuscript, FW participated in the design, TS IS AS JL participated in the surgery and data collection, FF participated in coordination and performed statistical analysis. All authors read and approved the final manuscript.
